# Is a Bacteriophage Approach for Musculoskeletal Infection Management an Alternative to Conventional Therapy?

**DOI:** 10.3390/life15101534

**Published:** 2025-09-29

**Authors:** Jörg Eschweiler, Christian Fischer, Filippo Migliorini, Johannes Greven, Thomas Mendel, Philipp Kobbe, Steffen Langwald

**Affiliations:** 1 Department of Trauma and Reconstructive Surgery, BG Klinikum Bergmannstrost Halle, 06112 Halle (Saale), Germany; christian.fischer@bergmannstrost.de (C.F.); thomas.mendel@bergmannstrost.de (T.M.); philipp.kobbe@bergmannstrost.de (P.K.); steffen.langwald@bergmannstrost.de (S.L.); 2 Department of Trauma and Reconstructive Surgery, University Hospital Halle, 06120 Halle (Saale), Germany; filippo.migliorini@uk-halle.de; 3 Innovationshub Muskuloskelettale Chirurgie Halle (IMCH), 06120 Halle (Saale), Germany; 4 Department of Orthopaedic and Trauma Surgery, Academic Hospital of Bolzano (SABES-ASDAA), 39100 Bolzano, Italy; 5 Department of Life Sciences, Health, and Health Professions, Link Campus University, 00165 Rome, Italy; 6 Department of Thoracic Surgery, University Hospital Aachen, RWTH Aachen University, 52074 Aachen, Germany; jgreven@ukaachen.de

**Keywords:** biofilm disruption, phage cocktails, multidrug-resistant bacteria, compassionate use, personalised medicine, regulatory challenges, adjunctive therapy

## Abstract

Antimicrobial resistance is a global threat to public health. The growing resistance of bacteria to commonly used antibiotics necessitates the search for and development of alternative treatments. Bacteriophage (or phage) therapy fits this trend perfectly. Phages that selectively infect and kill bacteria might represent, in some cases, the last therapeutic option. This overview provides case examples and discusses the potential development of phage therapy, examining its ethical and legal considerations in the context of current clinical practices. Additionally, it explores the advantages of utilizing phage products in patients for whom existing therapeutic options are limited or unavailable. Further clinical studies are necessary to broaden the understanding of phages, their dosage, and a standardised delivery system. These efforts are essential to ensure that phage-based therapy is not viewed as experimentation but as a routine medical treatment. Bacterial viruses are unlikely to become a miracle cure or a panacea for infections, but they may find an important role in medicine. Full legalisation of this treatment could help solve the problem of multidrug-resistant infectious diseases on a global scale.

## 1. Introduction

Antimicrobial resistance (AMR) is one of the greatest global threats to public health [1]. In 2019, there were an estimated 4.95 million deaths worldwide in connection with AMR, with at least 1.27 million deaths per year being directly attributable to AMR [2,3,4]. The burden of infections with multi-resistant pathogens (MRPs) in the European Union has increased significantly compared to other infectious diseases and has steadily gained importance since 2007 [5]. The problems caused by this are cost and time factors in patient isolation and the complication of therapeutic measures, as antibiotic classes relevant to therapy have become ineffective [4].

In Germany, 400,000 to 600,000 patients fall ill with nosocomial infections every year [2,6]. Furthermore, around 9650 people die each year from a disease caused by MRP throughout Germany, and 45,700 deaths occur in the context of AMR [2]. Without infections with AMR pathogens, almost half a million deaths could have been avoided in the G7 countries in 2019 [2]. The development of resistance in bacteria has thus become one of the greatest threats to public health worldwide, resulting in the drugs used for treatment, especially antibiotics, being less or no longer effective [4].

Orthopaedic and trauma surgery is associated with a relatively high risk of infection and is, therefore, potentially affected by any existing antibiotic resistance. Surgical fracture treatment and joint arthroplasty are life-improving surgical procedures for millions of people around the world [7]. In addition to the functional benefits that the implantation of osteosynthesis or endoprostheses can bring, there is a risk of infection for every implant in these procedures.

Infection radicalization following the formation of a chronic biofilm poses a particular clinical challenge [7]. Microbial resistance to commonly used antibiotics is on the rise worldwide, making the treatment of infectious diseases increasingly difficult. Innovative antibiotics with previously unused mechanisms of action are urgently needed to counteract this trend.

Given the increasing multidrug resistance to antibiotics, the approach of generating alternatives appears to be worthwhile in this case [8]. Bacteriophage or phage therapy represents a genuine alternative, highlighting the potential advantages of phages over antibiotics, such as host specificity and lower toxicity to humans from naturally occurring bacteria, e.g., in the intestine or microbiome. Phage therapy can improve the treatment of bone and implant-associated infections in AMR [9]. Phages are viruses that only infect and kill bacteria [10]. They are currently only approved as medication in some countries of the Eastern Bloc (e.g., Ukraine, Georgia, Russia, Poland) [11,12]. In Germany, therapy is only possible as part of individual treatment trials.

Phage therapy represents a significant global health aspect: the antibiotic crisis is described as extraordinary, as MRPs do not stop at borders. What makes them remarkable is their precision; unlike antibiotics, they target only harmful bacteria, leaving beneficial ones intact. As antibiotic resistance grows into a global health crisis, phage therapy might be the breakthrough we need.

This investigation aims to provide an overview of phage therapy and the benefits of this approach.

## 2. Background of Bacteriophages and Clinical Trials

### 2.1. Bacteriophage Biology

MRP and AMR are serious issues. Patients die from banal infections or ultimately sepsis caused by bacteria. Phage represents a promising treatment approach for infections or colonization by MRP.

Phages are viruses that specialize in attacking and destroying bacteria [10,13,14,15,16,17,18]. At around 50 to 200 nanometres, phages are many times smaller than bacteria and have a relatively simple structure [15]. Most known phages belong to the order Caudovirales and have a so-called head-tail structure. Based on their morphology, which is visible under an electron microscope, they are classically divided into three groups [15]. Myoviruses have a contractile, long tail, siphoviruses have a non-contractile, long tail, and podoviruses have a non-contractile, short tail [10,15]. The head serves to store DNA, while the tail is necessary for the specific recognition of the bacterial receptor. However, various other forms of phages use single-stranded DNA or RNA to store genetic information [10,15]. The size of the genome is approximately ten to a thousand times smaller than that of bacteria.

**Figure 1:**
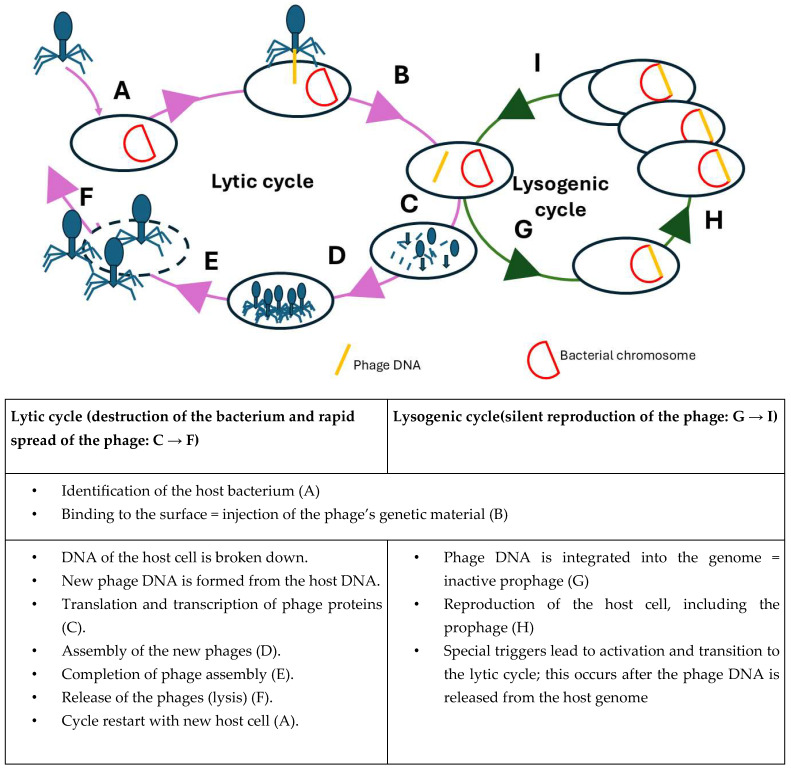
Bacteriophage entering a potential “enemy”.

These viruses infect and replicate only in bacterial cells. Phages can be used against biofilms as therapy or prophylaxis, either alone or in combination with antibiotics [13,14,19,20]. They offer a powerful, natural solution to infections. When phages infect a bacterium, they inject their genetic material (DNA or RNA) into the host cell (Figure 1) [21,22,23].

They can multiply in two possible ways: one is based on a lytic cycle (Figure 1) [24]. Here, the phage’s genetic material is read immediately, and a large number of phage nucleic acids and proteins are produced. Lytic phages rapidly infect, hijack the host’s cellular machinery to replicate, and then lyse the bacterial cell, releasing progeny phages [21,22,23]. This cycle is central to therapeutic applications because of its bactericidal nature and minimal risk of transferring harmful genes [25].

On the other hand, reproduction is based on a lysogenic cycle (Figure 1), in which the phage’s genetic material is incorporated into the bacterial genome [24]. As a result, when the bacterium multiplies, the phage’s genetic material is also replicated without damaging the host cell. Lysogenic phages integrate their genome into the bacterial chromosome, existing as a prophage and replicating passively with the host. Under certain conditions, they may switch to the lytic cycle. However, their potential to carry and disseminate virulence factors or antibiotic resistance genes makes them less desirable for clinical use.

One of the defining features of phages is their high specificity for their bacterial hosts [11,26]. A given phage typically infects only a narrow range of strains within a species. This specificity arises from the molecular recognition between phage tail fibers and bacterial surface receptors—proteins, polysaccharides, or other membrane components. This intimate interaction leads to a dynamic evolutionary arms race. Bacteria evolve defense mechanisms such as receptor mutation, CRISPR-Cas immunity, and restriction–modification systems. In response, phages evolve countermeasures, leading to a continual adaptation cycle. This co-evolutionary process makes phages highly adaptable but also necessitates continuous monitoring and development in therapeutic settings.

While the primary interest in phages lies in their ability to lyse bacteria, their interactions with hosts are far more nuanced. Some phages modulate bacterial metabolism, influence quorum sensing, or impact biofilm formation [27]. Certain phages can even suppress bacterial virulence or make bacteria more susceptible to antibiotics, offering synergistic possibilities in combination therapies.

Understanding their biology is essential for safely and effectively harnessing them as therapeutic agents.

### 2.2. Regulatory Framework and Aspects Regarding the Use of Phage Therapy

The history of bacteriophages dates back to the early 20th century, although there are hints of their existence prior to that time. The first person to isolate what we now call a bacteriophage was Frederick William Twort in 1915, in England, who observed an unusual filterable and infectious agent that could kill bacteria [28,29]. Shortly after, in 1917, Félix d’Hérelle independently discovered bacteriophages and coined the term “bacteriophage” [28].

Experimental phage therapy has been established for decades at the Eliava Institute of Bacteriophage, Microbiology, and Virology (EIBMV, established 1923) of the Georgian Academy of Sciences in Tbilisi (off-the-shelf fixed-phage products) and the Ludwik Hirschweld Institute of Immunology and Experimental Therapy (HIIET, established 1952) of the Polish Academy of Sciences in Poland (Wroclaw) [3,11,29]. Therapies carried out to date show no evidence of relevant phage therapy-associated side effects and are therefore initially harmless for the patient concerned [30].

The regulatory landscape surrounding phage therapy is complex, evolving, and uneven worldwide. As phage therapy reemerges as a potential answer to the antibiotic resistance crisis, its path to integration into clinical practice remains impeded by regulatory uncertainty, differences in national approaches, and the inherent biological variability of phages themselves. One of the central regulatory dilemmas concerns standardization versus personalization. Unlike conventional antibiotics, phages often need to be tailored to specific bacterial strains. Regulatory agencies such as the U.S. Food and Drug Administration (FDA) and the European Medicines Agency (EMA) typically require standardized, reproducible products for approval. This clashes with the inherently adaptive and individualized nature of phage therapy, especially when phage cocktails must be frequently updated to match evolving pathogens.

A second issue involves classification and legal definitions. Phages can be considered biological medicinal products, advanced therapy medicinal products, or even “living entities,” depending on jurisdiction. This lack of consensus complicates the approval process and creates uncertainty for developers. In the European Union, for example, phage therapy falls under complex biologics regulation, while in the United States, it is still managed within the framework of investigational new drugs (INDs).

Quality control and manufacturing standards present further obstacles. Phages are biological entities that require host bacteria for production, raising concerns about contamination with endotoxins, residual bacterial DNA, and other impurities. Ensuring compliance with Good Manufacturing Practice (GMP) standards is more complicated than for conventional small-molecule drugs. Additionally, questions about stability, storage, and shelf life make large-scale commercialization challenging.

### 2.3. Clinical Trials

The design of clinical trials is another sticking point. Traditional randomized controlled trials are considered the gold standard for drug approval, but their rigid structure does not easily accommodate the adaptive, patient-specific nature of phage therapy. This mismatch raises debates about alternative trial designs. Globally, there are regional disparities in regulatory acceptance. Countries such as Georgia and Poland have a long tradition of phage therapy and allow its clinical use, while most Western nations limit phages to research contexts or compassionate-use cases. This uneven regulatory landscape hampers international collaboration and discourages investment.

Developing therapeutics based on phage requires an investment in rigorous clinical trials (CTS) [31]. These CTS must be of the same design and scope as those that would be applied to the development of antibiotics [32].

The number of CTS registered in https://clinicaltrials.gov (accessed on 17 September 2025) that use phages has increased (Table 1). Public–private partnerships and non-profit consortia are funding early-phase clinical trials and quality manufacturing pipelines in the hope of establishing scalable models.

The table compiled from recent clinical trial registries provides a window into the evolving landscape of bacteriophage therapy research. The data captured in the table highlights both the promise and the challenges of phage therapy as it moves from concept to clinical practice.

One of the most striking features of the table is the diversity of medical conditions targeted. Trials explore the use of phages in urinary tract infections, tonsillitis, gastrointestinal disorders, and chronic or refractory infections. This spread reflects the inherent adaptability of phages: their capacity to be tailored to different bacterial pathogens makes them attractive candidates across a range of clinical contexts. In particular, the presence of trials in respiratory and urinary infections underscores the urgent need for alternatives to antibiotics in areas where resistance is widespread.

Equally important is the status of these trials. The table includes entries that are completed, ongoing, or in uncertain states. Completed trials suggest that efficacy and safety data may already exist, though the extent of publication and dissemination remains variable. Ongoing trials indicate active global efforts to test phages in controlled settings, while “unknown” or inactive trials reveal gaps in continuity, funding, or reporting. Taken together, the data emphasize that while phage therapy is gaining traction, its development pipeline is still relatively immature compared to that of conventional drugs.

The nature of the interventions described in the table further illustrates current trends in phage research. Some studies employ single phage formulations, while others use phage cocktails or combinations of phages with antibiotics. The inclusion of combined regimens reflects a growing recognition of phage–antibiotic synergy, whereby the two agents can complement each other in overcoming bacterial resistance and preventing treatment failure. This pragmatic approach may also increase the likelihood of regulatory approval, as it situates phages within the broader antimicrobial toolkit rather than as standalone replacements.

From a methodological perspective, the table reveals a mix of observational and interventional studies. Observational trials, such as the establishment of phage biobanks, serve an essential preparatory role by cataloging and characterizing phages for future use. Interventional studies, though fewer in number and often limited in scale, are critical for establishing clinical evidence of efficacy and safety. The limited prevalence of large, randomized controlled trials underscores one of the central regulatory challenges facing the field: the difficulty of adapting conventional trial designs to therapies that are inherently individualized and dynamic.

### 2.4. A Non-Standardized Global Framework

Unlike conventional pharmaceutical drugs, phages present unique challenges to regulators. Their high specificity, ability to evolve, and the necessity for individualized formulations clash with the standardized, one-size-fits-all models used by regulatory bodies such as the U.S. Food and Drug Administration (FDA) and the European Medicines Agency (EMA). Currently, there is no harmonized international framework for evaluating and approving phage therapies (see Section 2.5), which has led to divergent strategies: in the United States, phage therapy is largely experimental. While the FDA has granted compassionate use or expanded access exemptions for individual patients, there is no approved phage therapy product available on the market. Clinical trials are underway (see Table 1), but the regulatory pathway remains undefined and unpredictable.

In the European Union, the EMA has recognized the potential of phage therapy but similarly lacks a formalized route to approval. For example, Belgium, have taken proactive steps. Belgium’s creation of a “magistral phage” framework—allowing for the preparation of tailor-made phage medications in pharmacies—represents a progressive model for balancing safety with accessibility. The current problem with the use of phages is that therapy in Germany is currently only possible in the context of individual treatment trials [4].

In countries like Georgia and Russia, phage therapy has a long history and continues to be used routinely [28]. Here they are sold and used as over-the-counter medicines for treating and preventing various infectious diseases. They are produced there commercially by companies or in pharmacies as standardized “off-the-shelf” products. However, these practices are often not aligned with modern regulatory standards, particularly those concerning quality assurance and Good Manufacturing Practices (GMP).

Compounding these challenges is the issue of intellectual property: natural phages are difficult to patent, reducing incentives for pharmaceutical companies to invest in large-scale clinical development.

### 2.5. Challenges in Regulation

The regulatory process is complicated by several critical aspects: phage therapy often requires bespoke phage cocktails, particularly for drug-resistant infections where no off-the-shelf solution exists [4]. Traditional drug approval processes assume fixed formulations and are therefore ill-suited to this paradigm. While the rapid evolution of phages is advantageous in combating bacterial resistance, it poses challenges from a regulatory standpoint, where stability and consistency are paramount. Ensuring the sterility, purity, and potency of phage preparations is technically challenging. The lack of standardized protocols across laboratories and countries hinders scaling up and international cooperation [33].

Regulatory bodies are considering phage banks, pharmacovigilance systems, and platform technologies that could facilitate the approval process for adaptable phage cocktails [3,33].

In conclusion, the promise of phage therapy is tempered by a complex regulatory environment that struggles to reconcile the unique biological properties of phages with frameworks designed for conventional drugs. Moving forward, regulatory innovation will be as critical as scientific progress. Adaptive approval pathways, harmonized international standards, and a willingness to accept personalized treatment models may be necessary to unlock the full potential of phage therapy.

## 3. Therapy Strategies

### 3.1. Current Momentum and Emerging Solutions

Despite these challenges, momentum is building. The increasing threat of antibiotic resistance has brought phage therapy into the spotlight, prompting several recent initiatives aimed at addressing regulatory gaps [34]. To date, no phage preparations have been approved as medicinal products in the EU or the USA, and can only be used under regulatory exceptions for special situations of need [4,35].

### 3.2. Case Report

Included were three patients who could not be treated by prosthesis replacement and antibiotic therapy due to significant co-factors (e.g., bone stock, soft tissue situation, MRP, surgically non-remediable focus of infection). Each patient was treated as part of an individual healing trial with ready-made Georgian phage lysates, integrated into the surgical treatment concept. Information for each patient were presented in that way that no identification is possible to ensure data protection considering the German data regulation.

#### 3.2.1. Case 1—Proximal Femur

Infections after fracture can be challenging, particularly with concomitant severe bone defects and multi-resistant microorganisms. We present a case of a 43-year-old patient (male) with a fracture-related infection following an injury from a traffic accident, resulting in a subtrochanteric segmental bone defect and the detection of four different multi-resistant Gram-negative bacteria. Due to antibiotic drug resistance, treatment with phages was considered.

**Figure 2:**
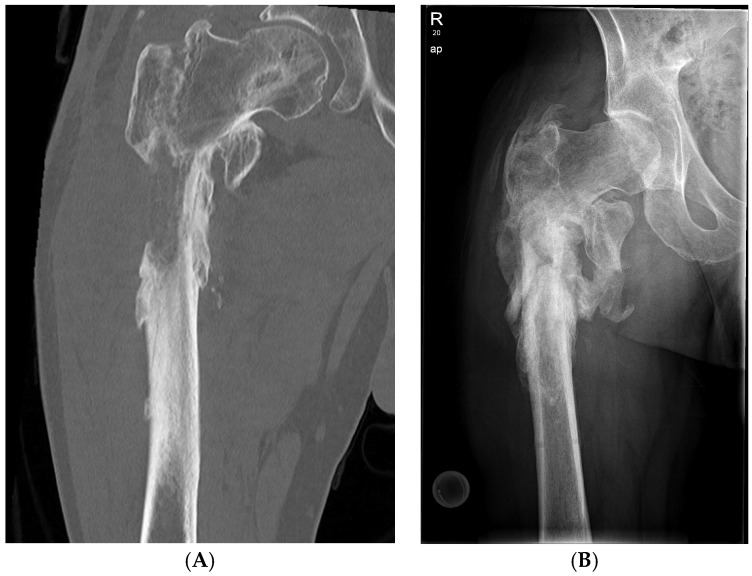
Phage treatment of a large femoral defect: (**A**) osteomyelitis situation with significant bone loss; (**B**) bone remodeling after phage therapy.

Diagnosis: multiple traumata sustained in a traffic accident in August 2023, including:

▪ Multi-fragmentary tibial shaft fracture on the left (AO 42 C3)
▪ Implant-associated infection and soft tissue defect on the lateral thigh on the right following open reduction and internal osteosynthesis of a multi-fragmentary subtrochanteric femur fracture (AO 31A2) on the right with locking nail and two cerclages in September 2023 (Figure 2)
▪ Subglottic stenosis after external tracheotomy and relocation of the tracheostoma ex domo
▪ Multiple rib fractures on both sides, 4th to 8th ribs
▪ Traumatic brain injury with ICB (intracranial bleeding)
▪ Fracture of the transverse process of the thoracic vertebra body (TVB) 7
▪ Clavicle fracture on the left
▪ Fracture of the lateral mass of the sacrum on the left
▪ Fracture of the anterior acetabular pillar on the left
▪ Following sacral decubitus and decubitus of the heel on the right

Phage susceptibility testing revealed the activity of a commercially available bacteriophage cocktail (Intesti bacteriophage, Eliava Institute, Tbilisi, Georgia). This phage cocktail was included in a modified two-stage Masquelet technique. During the first intervention, the bone was debrided, and samples for microbiological and phage testing were harvested.

The case presented here exemplifies the successful use of individualized bacteriophage therapy in the context of a multi-resistant soft tissue infection following open trauma. As in the present case, phage therapy is currently reserved for individual therapeutic trials due to the lack of drug approval. In this case, such a trial was justified due to the failure of established therapeutic methods to preserve the extremities.

#### 3.2.2. Case 2—Humerus

**Figure 3:**
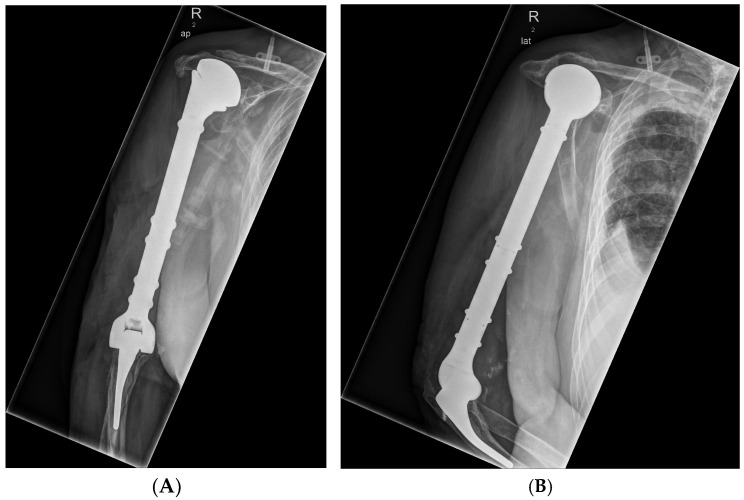
The situation after BP therapy (**A**) and treatment with a complex humerus implant (**B**).

The following case showed the results after an initial fall from a truck, resulting in a distal humeral comminuted fracture on the right side, which subsequently developed into infectious pseudarthrosis. In 2016, the patient was referred to our clinic due to the humeral displacement of the implant. After multiple surgical procedures, the proximal humerus was finally resected in 2017, and the implantation of a total humeral replacement on the right side with connection to the rotator cuff was done (Figure 3).

Diagnosis:

▪ Chronic periprosthetic infection of the implanted alloplastic humerus and elbow joint replacement on the right side with fistula
▪ Infection-related loosening of a modular elbow joint endoprosthesis with osteitis of the proximal humerus on the right side

Intraoperative samples were taken in preparation for treatment with bacteriophages. Colonisation with *Staphylococcus epidermidis*, *Staphylococcus capitis*, and *Cutibacterium acnes* was detected. Phage therapy combined with antibiotics was started with the following strategy:

Antibiotics:

21 February 2024 until 27 February 2024: Unacid 3 g intravenous, 3 times a day

21 February 2024 until 27 February 2024: Teicoplanin 400 mg intravenous, once a day

4 March 2024 until 7 March 2024: Unacid 3 g intravenous, 3 times a day

4 March 2024 until 7 March 2024: Teicoplanin 400 mg intravenous, once a day

7 March 2024 switch to: Amoclav pills 875 mg/125 mg, 3 times a day

Phages:

1 March 2024: SES Bakteriophage, 10 mL, once a day

4 March 2024 until 11 March 2024: SES Bacteriophage 10 mL, once a day

3 April 2024 until 9 April 2024: SES Bacteriophage 10 mL, once a day

After 11 days, intraoperative samples were taken again, but despite multiple representative samples, no pathogens could be detected. Thus, the previously used bacteriophages proved to be sufficiently effective against the aforementioned bacterial strains.

After completing the therapeutic measures, the patient could be discharged in stable general condition with a non-irritated and dry wound and soft tissue conditions.

#### 3.2.3. Case 3—Spine/Knee

The patient (female, 82 years) complained of a progressive deterioration in her general condition with pronounced myalgia in the shoulder and pelvic girdle, omalgia on the left, gonalgia on the right, and an increase in chronic oedema of the lower extremity. In addition, the patient complained of an increase in pre-existing pain in the lumbar spine. Laboratory tests showed significantly elevated inflammation values. An infection focus diagnosis was performed. Among other things, blood cultures were taken, which revealed Gram-positive cocci. In addition, a puncture was performed on the right knee joint and the left shoulder joint, in which Gram-positive cocci were also detected. Suspecting septic polyarthritis with septicemia, the patient was transferred to our facility.

**Figure 4:**
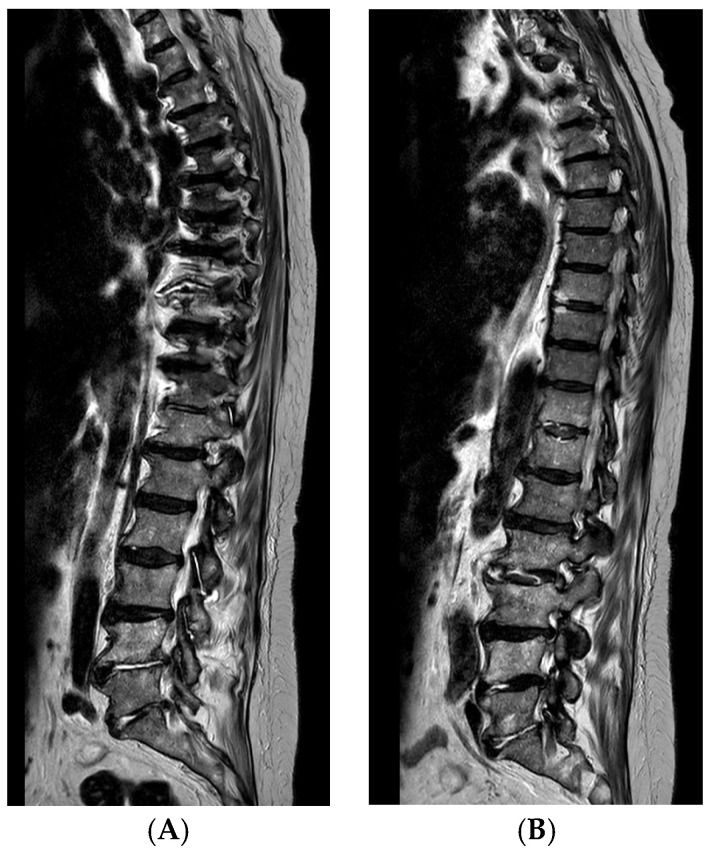
Multiple spondylodiscitis changes of the spine: (**A**) thoracic; (**B**) lumbar.

**Figure 5:**
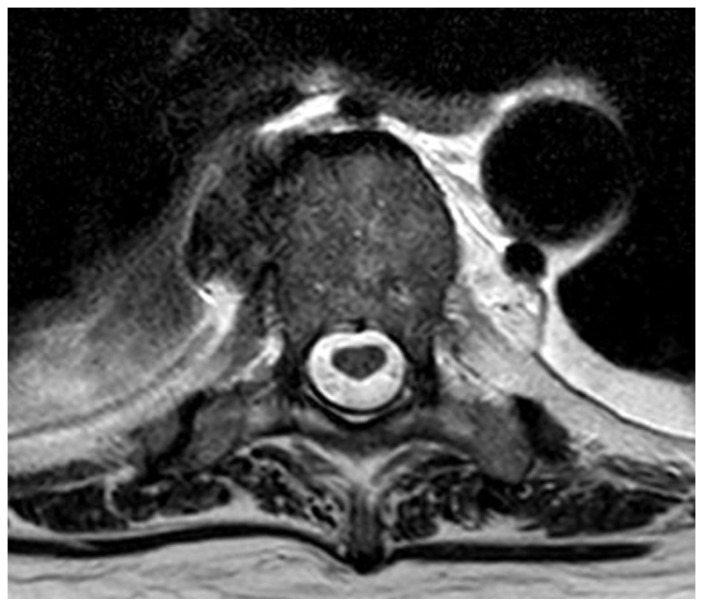
Spondylodiscitis changes.

Diagnosis:

▪ Multi-level spondylodiscitis in segments TVB 8/9, TVB 11/12, LVB (lumbal vertebra body) 2/3, and LVB 4-SVB (sacral vertebra body) 1 with inflammatory reaction and epidural abscess formation at the posterior edges of LVB 4 and LVB 5, as well as a long-distance, meningeal, inflammatory surrounding reaction at TVB 8–12 and LVB-2/SVB-1 with accompanying consecutive absolute spinal canal stenosis in the LVB 4–5 segment (Figure 4 and Figure 5)
▪ bilateral partially chambered psoas abscesses
▪ Epidural abscess at the posterior edges of LS (lumbal segment) 5 and LS6 with consecutive absolute spinal canal stenosis in the LS5/SS (sacral segment) 1 segment
▪ Shoulder joint empyema on the left
▪ Periprosthetic infection with an implanted revision total knee arthroplasty on the right
▪ Exclusion of periprosthetic infection with implanted total hip arthroplasty on the left

In consultation with the neurosurgeons and radiologists, a CT-guided drainage of the psoas abscesses with accompanying conservative antibiotic therapy for spondylodiscitis was realized. The anti-infective therapy was adjusted several times following the resistogram.

Under the strong suspicion of a periprosthetic infection of the existing revision total knee replacement, the possibility of phage therapy was discussed with the patient. Here, the therapy strategy with the phage was as follows:

10 January 2024 until 16 January 2024: Fersisi Bacteriophage 10 mL, oral, 2 times a day

18 January 2024 until 24 January 2024: Fersisi Bacteriophage 10 mL, oral, 2 times a day.

10 January 2024 until 16 January 2024: Fersisi Bacteriophage 10 mL, 1× intra-articular

application daily via the inserted lock on the right

knee joint (first cycle)

18 January 2024 until 24 January 2024: Fersisi Bacteriophage 10 mL, 1× intra-articular

application daily via the inserted lock on the right

knee joint (second cycle)

Throughout treatment, the inflammation values regressed sufficiently.

## 4. Discussion

From an overall perspective, it is conceivable that phage preparations, because of the increasing AMR, will be of great importance [36]. Phage therapy holds significant promise for treating resistant musculoskeletal infections, particularly in implant-related cases. In the global antibiotic crisis, phage therapy is an essential supplement to antibiotic therapy if patients are no longer able to tolerate antibiotics. The advantages of phages over antibiotics are their specific effect and the avoidance of dysbiosis. A further advantage is the lack of toxicity, the prevention of the selection of additional new antibiotic-resistant pathogens, and the self-regulating effect without accumulation in the body. Furthermore, phages can be engineered or selected for enhanced activity, host range expansion, or combined with antibiotics. Combining phages with antibiotics can produce synergistic effects, reducing the required dose of both agents and limiting the risk of resistance development.

### 4.1. Clinical Trials

Despite these promising attributes, the clinical use of phage therapy in musculoskeletal surgery is still largely experimental [37,38]. The absence of large-scale randomized CTS further limits evidence-based recommendations. Designing trials for phage therapy is difficult due to the specificity of phage-host interactions. Randomized controlled trials require homogeneous patient populations and standard treatments, conditions rarely met in real-world phage applications. Also, the question of how the use of phage products in clinical trials with the aim of their production and approval as medicinal products in Western countries (e.g., in the EU or the USA) can be organized must be discussed.

In addition, much of the evidence for phage therapy is derived from a minimal number of phage isolates, slowing the global acceptance of phage as a therapeutic. Of the 1031 existing phages, fewer than 104 have been isolated and sequenced to date [7].

The development of rigorous and reproducible laboratory techniques that predict clinical activity of phages is still in its infancy and must also be prioritized as clinical investigations proceed [32]. In addition, the routine and timely extraction of phages, and the production of phage cocktails under good manufacturing practice conditions must be considered a challenge.

**Figure 6:**
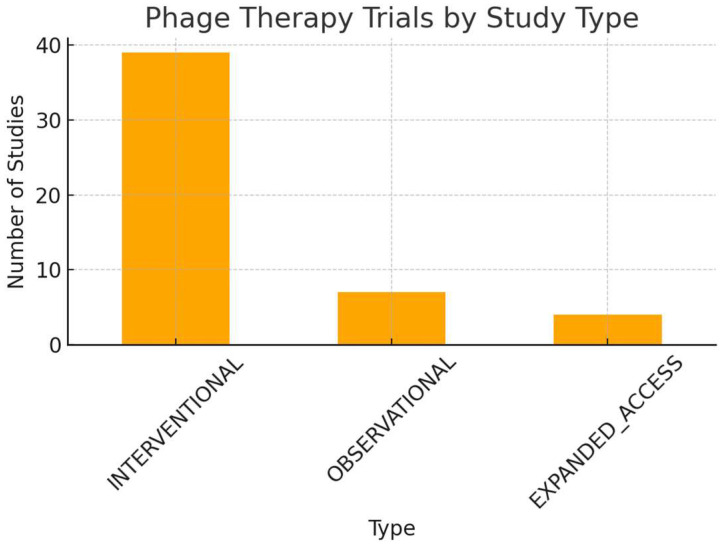
Shows the distribution by study type (interventional vs. observational).

The table of clinical trials (Table 1) illustrates a field that is vibrant but still in a formative stage. Phage therapy is being tested in diverse infections, with innovative strategies that often involve combination therapies. Yet, the modest scale of many studies, the variability in trial status, the variability in study type (Figure 6), and the continuing lack of standardized regulatory frameworks highlight the road ahead.

If phage therapy is to realize its potential as a response to the global antibiotic resistance crisis, the exploratory efforts reflected in Table 1 will need to be consolidated into rigorous, large-scale, and internationally coordinated clinical programs.

### 4.2. Regulatory Affairs

The regulatory framework for phage therapy is at a crossroads [35]. While there is growing recognition of its therapeutic potential, progress remains contingent on flexible, science-based regulatory innovation. Harmonizing standards, adopting adaptive models, and acknowledging the personalized nature of phage therapy will be crucial steps in bringing this century-old approach into 21st-century medicine. Continued dialogue between researchers, regulators, clinicians, and patients is essential to ensure that phage therapy becomes a safe, effective, and accessible tool in the global fight against AMR.

### 4.3. Case Reports

All of the presented cases showed successful treatment. Throughout treatment, laboratory tests showed a decline in inflammation values and a significant improvement in the patient’s general condition. In case 1, the contribution of phage therapy to the patient’s rapid and impressive improvement cannot be objectively determined. The effectiveness of this approach in this case is supported by the patient’s remarkable improvement. Secondly, in the follow-up procedure, 17 days after the initial procedure and 7 days after the end of local phage application, the findings were macroscopically completely infection-free, except for a tiny subcutaneous abscess that had to be excised in toto.

In case 2, a rescue of the upper arm could be realized with the combination of surgical renovation and phage therapy.

In Case 3, the patient left the hospital in stable general condition with dry and non-irritated wounds and no further signs of infection. In sum, phage showed a good performance in the treatment of infections and a positive benefit for the treated patients, respectively.

Phages have several advantages over antibiotics. Phages specifically attack only one target bacterium, leaving the normal microbiome untouched. They are well-tolerated and have no serious side effects [39,40]. However, treating infections with phages and antibiotics together can bring further benefits. The mere presence of phages can, for example, restore the sensitivity of resistant bacteria, allowing antibiotics to become effective again [41,42].

### 4.4. Limitations of Phage Therapy

Unlike antibiotics, phage therapy lacks standardized dosing, administration routes, and regulatory guidelines [4,5]; additionally, variability in phage preparation (e.g., purity, titer, and stability) complicates clinical translation. However, while the potential is exciting, several scientific, logistical, and regulatory bottlenecks must be resolved before it becomes a mainstream treatment. The host spectrum of phage also speaks in favor of this. The specificity of bacteriophages, while a benefit, also poses a limitation: a phage that is highly effective against one strain of bacteria may be completely ineffective against another. This is in contrast to antibiotics, which usually have a broad host spectrum and can therefore cause collateral damage in the body, e.g., in the microbiome. However, until then, it remains a promising but investigational tool, best used in combination with antibiotics and surgical debridement.

Therefore, precise microbiological identification and susceptibility testing are required, which may delay treatment initiation. Challenges include the minimal availability of phages and a lack of testing infrastructure, which makes it challenging to apply phages on time following phagograms.

Furthermore, there is a lack of lytic phages for certain bacterial pathogens, a lack of development of antibodies for phages, and endotoxin release.

One significant hurdle in phage therapy is the absence of suitable lytic phages for all bacterial pathogens, particularly obligate intracellular bacteria such as *Rickettsia*, *Ehrlichia*, and *Coxiella* [43,44]. These bacteria reside and replicate within host cells, presenting a unique challenge for phages that typically target extracellular bacteria. While these pathogens may not pose a widespread public health threat, they can cause severe and even fatal diseases, such as ehrlichiosis [43]. The primary mechanism of phage therapy relies on lytic phages that can efficiently infect and destroy bacterial cells [17,40,45]. For obligate intracellular bacteria, phages must overcome several barriers to reach their bacterial targets [23]. This cellular entry is a complex process that is not naturally inherent to most phages. Current research is exploring strategies such as engineering phages with cell-penetrating peptides to facilitate their entry into host cells and subsequent targeting of intracellular bacteria [46]. However, these approaches are still in experimental stages and are not yet widely available or clinically established solutions. The inherent specificity of phages, while an advantage in avoiding harm to beneficial microbiota, also means that a specific lytic phage must be identified or engineered for each target pathogen, a task that remains unfulfilled for many important intracellular bacteria [46].

The human immune system’s response to phages represents another significant limitation in phage therapy [47]. Patients undergoing phage therapy can develop antiphage antibodies, which are immune proteins that recognize and neutralize phages [48,49]. The clinical implications of these antibodies are still being investigated, but they have the potential to reduce the efficacy of phage therapy by promoting the rapid clearance of phages from the body [50,51]. Phage–antibody complexes could potentially accumulate in tissues and organs, leading to inflammation, injury, or other late complications [48]. Therefore, careful monitoring of antibody levels in patients receiving phage therapy is considered prudent, and in cases where antibody levels rise significantly, discontinuation of therapy might be necessary. The kinetics of antibody disappearance can vary considerably among individuals, with some patients exhibiting elevated levels for over a year after treatment [50,52]. This variability underscores the complexity of the immune response to phages and highlights the need for further research to fully understand its impact on both the safety and effectiveness of phage therapy.

One of the immediate and potentially severe side effects of phage therapy, particularly when treating infections caused by Gram-negative bacteria, is the rapid and massive breakdown of bacterial cells, leading to the release of bacterial components, including endotoxins [24]. Endotoxins are potent inducers of inflammatory cytokine responses in the host, and their sudden release can trigger a range of adverse reactions [24,53]. While the rapid bacterial lysis is the desired therapeutic effect, the subsequent release of endotoxins poses a challenge that requires careful management. Beyond endotoxins, other bacterial components such as bacterial DNA, enterotoxins, exotoxins, or lipoteichoic acid (from Gram-positive bacteria) can also be present in phage preparations and contribute to adverse reactions [49]. The presence and concentration of these contaminants can significantly influence the safety profile of phage therapy. Therefore, rigorous purification of phage preparations is paramount to minimize the presence of these bacterial components and reduce the risk of severe adverse events, ensuring a safer therapeutic experience for patients.

### 4.5. Outlook

Novel delivery methods for orthopaedic and trauma applications could be the next innovative step to advance this approach. Here, new administration routes are being explored, including phage-coated implants (e.g., titanium prostheses with embedded phages), local hydrogels and bone cement (sustained-release formulations for post-surgical infection prevention), and intra-articular or intraosseous injections for deep infections. Phage therapy offers new perspectives, promising research ideas, and innovative functionalized implants.

Even if there are still gaps in our knowledge today, for example, regarding the behavior of phages in the human body, our understanding of the biological properties relevant to their use has improved considerably since the early applications. Given increasing antibiotic multi-resistance, the approach of using the natural enemies of bacteria therapeutically seems obvious and promising. Phage therapy is not yet a silver bullet, but it represents a paradigm shift in treating resistant musculoskeletal infections. The biggest breakthroughs will come from (1) improved phage engineering (broader spectrum, reduced immunogenicity), (2) standardized regulatory pathways for phage-based biologics, and (3) more robust clinical trials proving efficacy in orthopaedic and trauma infections. If these challenges are addressed, phage therapy could become a cornerstone of infection management in musculoskeletal surgery within the next decade.

## 5. Conclusions

The story of bacteriophages and phage therapy is one of early enthusiasm, decline, neglect, and now a renewed hope. From Twort and d’Hérelle’s pioneering work in the 1910s, through decades of Eastern European and Soviet practice, to the current age of antibiotic resistance, phages are increasingly viewed not just as scientific curiosities but as credible tools for treating bacterial infections. The historical record suggests that success depends heavily on rigorous characterization, matched phage–bacteria pairs, and proper regulatory and clinical frameworks.

In the treatment of infections, e.g., following hip and knee arthroplasty, or after trauma and reconstructive surgery, bacteriophages, whether used alone or in combination as cocktail therapy, have shown therapeutic potential. It should be taken into account that preoperative evaluation is essential, and appropriate phage types and treatment regimens must be selected based on bacteriological evidence [18].

## Figures and Tables

**Table 1:** Overview of CTS of phage therapy reported at https://clinicaltrials.gov (Condition/disease: Bacteriophage Therapy). The registry was queried using the keyword “bacteriophage therapy” on 17 September 2025.

NCT No.	Study Title	Study Status	Conditions	Interventions	Study Type
NCT05314426	Mayo Clinic Phage Program Biobank	ENROLLING_BY_INVITATION	Bacteriophage Therapy		OBSERVATIONAL
NCT05973721	Clinical Study of Phage Therapy for Chronic Constipation Efficacy and Safety	UNKNOWN	Pib Specific Phage|Intractable Constipation	BIOLOGICAL: phage	INTERVENTIONAL
NCT03140085	Bacteriophages for Treating Urinary Tract Infections in Patients Undergoing Transurethral Resection of the Prostate	COMPLETED	Intravesical Bacteriophage Treatment for Urinary Tract Infections	BIOLOGICAL: PYO Phage|DRUG: Antibiotics|OTHER: Sterile bacteriology media	INTERVENTIONAL
NCT04682964	Bacteriophage Therapy in Tonsillitis	ACTIVE_NOT_RECRUITING	Acute Tonsillitis	DRUG: Nebulizer inhalation irrigation of the mucous membranes of the tonsils with a bacteriophage.	INTERVENTIONAL
NCT06814756	Bacteriophage Therapy for Morganella Morganii Prosthetic Joint Infection	ACTIVE_NOT_RECRUITING	Prosthetic Joint Infections of Hip	BIOLOGICAL: phage therapy	INTERVENTIONAL
NCT04287478	Bacteriophage Therapy in Patients With Urinary Tract Infections	TERMINATED	Urinary Tract Infection Bacterial	BIOLOGICAL: Bacteriophage Therapy	INTERVENTIONAL
NCT05498363	Bacteriophage Therapy of Difficult-to-treat Infections	COMPLETED	Bacterial Infections	BIOLOGICAL: Bacteriophage therapy	OBSERVATIONAL
NCT04787250	Bacteriophage Therapy in Patients With Prosthetic Joint Infections	WITHDRAWN	Prosthetic Joint Infection	BIOLOGICAL: Phage Therapy|PROCEDURE: Two-Stage Exchange Arthroplasty	INTERVENTIONAL
NCT00945087	Experimental Phage Therapy of Bacterial Infections	UNKNOWN	Bacterial Infections	OTHER: Bacteriophage preparation	INTERVENTIONAL
NCT05537519	Phage Therapy for the Treatment of Urinary Tract Infection	ACTIVE_NOT_RECRUITING	Recurrent Urinary Tract Infection	BIOLOGICAL: Phage Therapy	INTERVENTIONAL
NCT06409819	Phage Therapy for Recurrent UTIs in Kidney Transplant Recipients	RECRUITING	Urinary Tract Infection, Recurrent	DRUG: phage therapy|DRUG: control	INTERVENTIONAL
NCT04803708	Bacteriophage Therapy TP-102 in Diabetic Foot Ulcers	COMPLETED	Diabetic Foot Ulcer|*Pseudomonas Aeruginosa* Infection|*Staphylococcus Aureus* Infection|Acinetobacter Infection	BIOLOGICAL: TP-102	INTERVENTIONAL
NCT05177107	Bacteriophage Therapy in Patients With Diabetic Foot Osteomyelitis	TERMINATED	Osteomyelitis|Diabetic Foot Osteomyelitis	BIOLOGICAL: Bacteriophage Therapy|OTHER: Placebo	INTERVENTIONAL
NCT06456424	Bacteriophage Therapy for Methicillin-Sensitive *Staphylococcus Aureus* Prosthetic Joint Infection	ACTIVE_NOT_RECRUITING	Prosthetic Joint Infections of Hip|*Staphylococcus Aureus* Infection	BIOLOGICAL: Phage therapy	INTERVENTIONAL
NCT05269134	Bacteriophage Therapy in Patients With Prosthetic Joint Infections (PJI)	WITHDRAWN	Prosthetic Joint Infection	DRUG: Bacteriophage|DRUG: Placebo	INTERVENTIONAL
NCT06559618	Bacteriophage Therapy in Spinal Cord Injury Patients With Bacteriuria	RECRUITING	Bacteriuria|Spinal Cord Injuries|Asymptomatic Bacteriuria|*Escherichia Coli*	DRUG: Phage Therapy|OTHER: Placebo	INTERVENTIONAL
NCT05269121	Bacteriophage Therapy in First Time Chronic Prosthetic Joint Infections	WITHDRAWN	Prosthetic Joint Infection|Bacterial Infections	BIOLOGICAL: Phage Therapy	INTERVENTIONAL
NCT06942624	Phage Therapy for the Treatment of a Chronic *Enterococcus Faecium* Periprosthetic Joint Infection	NOT_YET_RECRUITING	Periprosthetic Joint Infection	BIOLOGICAL: Phage Therapy	INTERVENTIONAL
NCT06827041	Use of Phage Therapy for Treatment of a Periprosthetic Joint Infection	ACTIVE_NOT_RECRUITING	Periprosthetic Joint Infection	BIOLOGICAL: Phage (Cytophage Technologies)	INTERVENTIONAL
NCT07048704	Taking Advantage of Phage Technologies (TAPT) to Facilitate Phage Therapy While Reducing the Use of Antibiotics in the Management of Cystic Fibrosis (CF)	NOT_YET_RECRUITING	Cystic Fibrosis (CF)|*Klebsiella Pneumoniae* Infection|*E Coli* Infections|*Staphylococcus Aureus* Infection|Achromobacter|*Stenotrophomonas Maltophilia* Infection	DRUG: Intravenous Bacteriophage Cocktail plus Standard IV Antibiotics	INTERVENTIONAL
NCT04684641	CYstic Fibrosis bacterioPHage Study at Yale (CYPHY)	COMPLETED	Cystic Fibrosis	DRUG: Standard Dose YPT-01|OTHER: Placebo	INTERVENTIONAL
NCT05948592	Bacteriophage Therapy TP-102 in Patients With Diabetic Foot Infection	RECRUITING	Diabetic Foot Infection	BIOLOGICAL: TP-102|OTHER: Placebo	INTERVENTIONAL
NCT06368388	Bacteriophage Therapy for Difficult-to-treat Infections: the Implementation of a Multidisciplinary Phage Task Force	RECRUITING	Musculoskeletal Infection|Chronic Rhinosinusitis (Diagnosis)|Sepsis|Pulmonary Infection|Hidradenitis Suppurativa	OTHER: Prospective data collection|OTHER: Prospective data collection	OBSERVATIONAL
NCT03395743	Individual Patient Expanded Access for AB-PA01, an Investigational Anti-*Pseudomonas Aeruginosa* Bacteriophage Therapeutic	NO_LONGER_AVAILABLE		BIOLOGICAL: AB-PA01	EXPANDED_ACCESS
NCT03395769	Individual Patient Expanded Access for AB-SA01, an Investigational Anti-*Staphylococcus Aureus* Bacteriophage Therapeutic	NO_LONGER_AVAILABLE		BIOLOGICAL: AB-SA01	EXPANDED_ACCESS
NCT07076238	Biomarker Investigation of Response to Bacteriophage Treatment for Bacterial Infection	RECRUITING	Nontuberculous Mycobacterial Lung Disease	BIOLOGICAL: Bacteriophage Treatment	OBSERVATIONAL
NCT04815798	Phage Therapy for the Prevention and Treatment of Pressure Ulcers.	UNKNOWN	Pressure Ulcer	COMBINATION_PRODUCT: Bacteriophage-loaded Microcapsule Spray|COMBINATION_PRODUCT: Placebo|PROCEDURE: Standard of Care	INTERVENTIONAL
NCT05369104	Phage Therapy in Prosthetic Joint Infection Due to *Staphylococcus Aureus* Treated With DAIR.	UNKNOWN	Infection of Total Hip Joint Prosthesis|Infection of Total Knee Joint Prosthesis	BIOLOGICAL: Anti-Staphylococcus aureus Bacteriophages	INTERVENTIONAL
NCT06870409	Bacteriophages in Addition to Antibiotics for the Treatment of Patients With Infective Endocarditis	RECRUITING	Endocarditis, Bacterial	DRUG: Bacteriophage	INTERVENTIONAL
NCT05010577	Nebulized Bacteriophage Therapy in Cystic Fibrosis Patients With Chronic *Pseudomonas Aeruginosa* Pulmonary Infection	COMPLETED	Chronic *Pseudomonas Aeruginosa* Infection|Cystic Fibrosis	DRUG: BX004-A|DRUG: Placebo	INTERVENTIONAL
NCT04650607	Phage Safety Cohort Study	RECRUITING	Prosthetic Joint Infection|Severe Infection	OTHER: Adverse event after injection of phages	OBSERVATIONAL
NCT06798168	Bacteriophage Clinical Trial for Periprosthetic Joint Infection of Multidrug Resistant *Pseudomonas Aeruginosa*	AVAILABLE	Joint Infection	BIOLOGICAL: Combining bacteriophage therapy with antibiotics for a case with hip PJI	EXPANDED_ACCESS
NCT04323475	Phage Therapy for the Prevention and Treatment of Wound Infections in Burned Patients	UNKNOWN	Wound Infection	BIOLOGICAL: Bacteriophage cocktail spray|DRUG: Xeroform	INTERVENTIONAL
NCT06185920	PHAGEinLYON Clinic Cohort Study: a Descriptive Study of Severe Infections Treated With Phage Therapy at the HCL.	RECRUITING	Severe Infection	OTHER: Description of severe infection	OBSERVATIONAL
NCT02664740	Standard Treatment Associated With Phage Therapy Versus Placebo for Diabetic Foot Ulcers Infected by S. Aureus	UNKNOWN	Diabetic Foot|Staphylococcal Infections	DRUG: Topical anti-Staphylococcus bacteriophage therapy|DRUG: Topical placebo corresponding to anti-Staphylococcus bacteriophage therapy	INTERVENTIONAL
NCT05967130	Treatment Chronic UTI Post Kidney Transplant	TERMINATED	Urinary Tract Infections|Transplant-Related Disorder	BIOLOGICAL: Phage	INTERVENTIONAL
NCT05616221	Study to Evaluate the Safety, Phage Kinetics, and Efficacy of Inhaled AP-PA02 in Subjects With Non-Cystic Fibrosis Bronchiectasis and Chronic Pulmonary *Pseudomonas Aeruginosa* Infection	COMPLETED	Non-cystic Fibrosis Bronchiectasis|*Pseudomonas Aeruginosa*|Lung Infection	BIOLOGICAL: AP-PA02|OTHER: Placebo	INTERVENTIONAL
NCT02116010	Evaluation of Phage Therapy for the Treatment of *Escherichia Coli* and *Pseudomonas Aeruginosa* Wound Infections in Burned Patients	UNKNOWN	Wound Infection	DRUG: *E. coli* Phages cocktail|DRUG: Standard of care: Silver Sulfadiazine|DRUG: P. Aeruginosa, Phages cocktail	INTERVENTIONAL
NCT06605651	Proof of Concept Study to Assess Safety and Efficacy of Phage Therapy in Hip or Knee Prosthetic Joint Infections Due to *Staphylococcus Aureus* Treated by DAIR.	NOT_YET_RECRUITING	Hip Prosthesis Infection|Knee Prosthesis Infection	BIOLOGICAL: Anti-*Staphylococcus aureus* Bacteriophages (PP1493 and PP1815) intra-articular injection with 0.9% NaCl solution|DRUG: 0.9% NaCl solution	INTERVENTIONAL
NCT06998043	Study With Phage for CF Subjects With Pseudomonas Lung Infection	RECRUITING	Chronic *Pseudomonas Aeruginosa* Infection|Cystic Fibrosis (CF)	BIOLOGICAL: BX004|OTHER: Placebo	INTERVENTIONAL
NCT05453578	A Phase 1b/2 Trial of the Safety and Microbiological Activity of Bacteriophage Therapy in Cystic Fibrosis Subjects Colonized With *Pseudomonas Aeruginosa*	COMPLETED	Bacterial Disease Carrier|Cystic Fibrosis	OTHER: Placebo|BIOLOGICAL: WRAIR-PAM-CF1	INTERVENTIONAL
NCT04636554	Personalized Phage Treatment in COVID-19 Patients With Bacterial Co-Infections Microbials for Pneumonia or Bacteremia/Septicemia	NO_LONGER_AVAILABLE	COVID-19|Bacteremia|Septicemia|Acinetobacter Baumannii Infection|*Pseudomonas Aeruginosa* Infection|Staph Aureus Infection	OTHER: Phage Therapy	EXPANDED_ACCESS
NCT00937274	Antibacterial Treatment Against Diarrhea in Oral Rehydration Solution	TERMINATED	Diarrhea	OTHER: T4 phage cocktail test|OTHER: Commercial T4 phage cocktail|OTHER: standard oral rehydration solution (ORS)	INTERVENTIONAL
NCT06370598	Phase 1/2a to Assess the Safety and Tolerability of TP-122A for the Treatment of Ventilator-Associated Pneumonia	NOT_YET_RECRUITING	Pneumonia, Ventilator-Associated	BIOLOGICAL: TP-122A	INTERVENTIONAL
NCT05488340	A Study of LBP-EC01 in the Treatment of Acute Uncomplicated UTI Caused by Drug Resistant *E. coli* (ELIMINATE Trial)	RECRUITING	Urinary Tract Infections	DRUG: LBP-EC01 0.1 × IV dose|DRUG: LBP-EC01 0.01 × IV Dose|DRUG: LBP-EC01 IV Infusion Dose|DRUG: Placebo|DRUG: LBP-EC01|DRUG: TMP/SMX	INTERVENTIONAL
NCT04596319	Ph 1/2 Study Evaluating Safety and Tolerability of Inhaled AP-PA02 in Subjects With Chronic *Pseudomonas Aeruginosa* Lung Infections and Cystic Fibrosis	COMPLETED	Cystic Fibrosis|*Pseudomonas Aeruginosa*|Pseudomonas|Lung Infection|Lung Infection Pseudomonal	BIOLOGICAL: AP-PA02|OTHER: Placebo	INTERVENTIONAL
NCT06262282	Mycobacteriophage Treatment of Non-tuberculosis Mycobacteria	ENROLLING_BY_INVITATION	Cystic Fibrosis|Nontuberculous Mycobacterial Lung Disease|Nontuberculous Mycobacterium Infection|Mycobacterium Infections|Mycobacterium; Pulmonary	BIOLOGICAL: mycobacteriophage	OBSERVATIONAL
NCT05184764	Study Evaluating Safety, Tolerability, and Efficacy of Intravenous AP-SA02 in Subjects With S. Aureus Bacteremia	COMPLETED	Bacteremia|*Staphylococcus Aureus*|*Staphylococcus Aureus* Bacteremia|Bacteremia Staph|Bacteremia Due to *Staphylococcus Aureus*	BIOLOGICAL: AP-SA02|OTHER: Placebo	INTERVENTIONAL
NCT06750588	Safety and Tolerability of NTR-101 in Patients With Acute Alcohol-Associated Hepatitis	NOT_YET_RECRUITING	Alcohol-Associated Hepatitis	DRUG: bacteriophage preparation|DRUG: Bacteriophage preparation|DRUG: bacteriophage preparation|DRUG: bacteriophage preparation	INTERVENTIONAL
NCT06319235	Clinical Trial to Demonstrate the Safety and Efficacy of DUOFAG^®^	RECRUITING	Surgical Site Infection|*Staphylococcus Aureus* Infection|*Pseudomonas Aeruginosa* Infection|Bacterial Infections|Surgical Wound Infection	DRUG: IMP|DRUG: Placebo	INTERVENTIONAL

## Data Availability

The datasets presented in this article are not readily available because of data protection. Requests to access the datasets should be directed to the corresponding author.

## Abbreviations

The following abbreviations are used in this manuscript:

AMR	antimicrobial resistance
AO	Arbeitsgemeinschaft Osteosysnthese
BP	Bacteriophage
CTS	Clinical Trials
DNA	Desoxyribonukleidacid
ICB	Intracranial bleeding
LVB	Lumbal vertebra body
LS	lumbal segment
MRP	multi-resistant pathogens
FDA	Food and Drug Administration
EMA	European Medicines Agency
RNA	Ribonukleidacid
SVB	Sacral vertebra body
SS	Sacral segment
TVB	Thoracic vertebra body
WHO	World Health Organization
